# Intranasal Delivery of Darunavir-Loaded Mucoadhesive In Situ Gel: Experimental Design, In Vitro Evaluation, and Pharmacokinetic Studies

**DOI:** 10.3390/gels8060342

**Published:** 2022-05-30

**Authors:** Anroop B. Nair, Sunita Chaudhary, Hiral Shah, Shery Jacob, Vivek Mewada, Pottathil Shinu, Bandar Aldhubiab, Nagaraja Sreeharsha, Katharigatta N. Venugopala, Mahesh Attimarad, Jigar Shah

**Affiliations:** 1 Department of Pharmaceutical Sciences, College of Clinical Pharmacy, King Faisal University, Al-Ahsa 31982, Saudi Arabia; baldhubiab@kfu.edu.sa (B.A.); sharsha@kfu.edu.sa (N.S.); kvenugopala@kfu.edu.sa (K.N.V.); mattimarad@kfu.edu.sa (M.A.); 2 Department of Pharmaceutics, Arihant School of Pharmacy & BRI, Adalaj, Gandhinagar 382421, India; sunita.pharmacy@swarrnim.edu.in (S.C.); hiral.pharamcy@swarrnim.edu.in (H.S.); 3 Department of Pharmaceutical Sciences, College of Pharmacy, Gulf Medical University, Ajman 4184, United Arab Emirates; dr.sheryjacob@gmu.ac.ae; 4 Department of Pharmaceutics, Institute of Pharmacy, Nirma University, Ahmedabad 382481, India; 20ftphdp67@nirmauni.ac.in; 5 Department of Biomedical Sciences, College of Clinical Pharmacy, King Faisal University, Al-Ahsa 31982, Saudi Arabia; spottathail@kfu.edu.sa; 6 Department of Pharmaceutics, Vidya Siri College of Pharmacy, Off Sarjapura Road, Bangalore 560035, India; 7 Department of Biotechnology and Food Science, Faculty of Applied Sciences, Durban University of Technology, Durban 4000, South Africa

**Keywords:** NeuroAIDS, darunavir, nasal in situ gel, mucoadhesive, brain targeting, optimization

## Abstract

The clinical efficacy of antiretroviral therapy in NeuroAIDS is primarily limited by the low perfusion of the drug to the brain. The objective of the current investigation was to design and develop an in situ mucoadhesive gel loaded with darunavir to assess the feasibility of brain targeting through the intranasal route. Preliminary batches (F1–F9) were prepared and evaluated for various pharmaceutical characteristics. A full factorial design of the experiment was applied to optimize and assess the effect of two influencing variables (Carbopol 934P (X_1_) and Poloxamer 407 (X_2_)) on the response effects (gelation temperature (Y_1_) and % drug release (Y_2_) at 8 h). The data demonstrate that both influencing variables affect the response variables significantly (*p* < 0.05). The optimized formulation (F7) exhibited favorable rheological properties, adequate mucoadhesion, sustained drug release, and greater permeation across the nasal mucosa. An in vitro ciliotoxicity study confirms the nontoxicity of the optimized in situ gel (D7) on the nasal mucosa. An in vivo pharmacokinetic study in rats was performed to assess drug targeting to the brain following the nasal application of the selected in situ gel (D7). Significantly higher (*p* < 0.0001) C_max_ (~4-fold) and AUC_0-α_ (~3.5-fold) values were noticed in the brain after nasal application, as compared to the intravenous route. However, less systemic exposure to darunavir was noticed with nasal therapy, which confirms the low absorption of the drug into the central compartment. Overall, the data here demonstrate that the optimized in situ mucoadhesive nasal gel is effective in targeting darunavir to the brain by the nasal route and could be a viable option for the treatment of NeuroAIDS.

## 1. Introduction

Acquired Immunodeficiency Syndrome (AIDS) has been one of the most distressing diseases caused by the Human Immunodeficiency Virus (HIV) in the last few decades [1]. Unfortunately, in more than half of HIV-positive people, neurological problems have been discovered. These neurological problems impact the central nervous system and the peripheral nervous system, or both [2]. Neuropsychiatric problems have been documented in HIV-positive and also in AIDS patients in recent years. NeuroAIDS refers to the neuropsychiatric problems that affect 30–50 percent of AIDS patients. The entry of HIV into the central nervous system via direct penetration across the blood–brain barrier or via peripherally contaminated macrophages is the primary cause of neuroAIDS [3]. Antiretroviral medication is an important part of the treatment of HIV/AIDS patients. On the other hand, even with combination antiretroviral therapy, neuroAIDS or HIV-linked neuronal abnormalities remain an important public health problem amongst AIDS patients. This is primarily because of the low perfusion of antiretroviral drugs to the brain, causing an insufficient drug level at the target, which is primarily limited by the formidable physiological blood–brain barrier [4]. Tight junctions in the endothelial cells of blood capillaries that lead to the brain operate as a barrier to most medications, preventing drugs and solutes from passing through [5]. As a result, even when using antiretroviral drugs for a long time, modest and persistent viral replication occurs in the central nervous system (CNS). Hence, new techniques to improve anti-HIV medication delivery to the CNS are needed.

**Figure 1:**
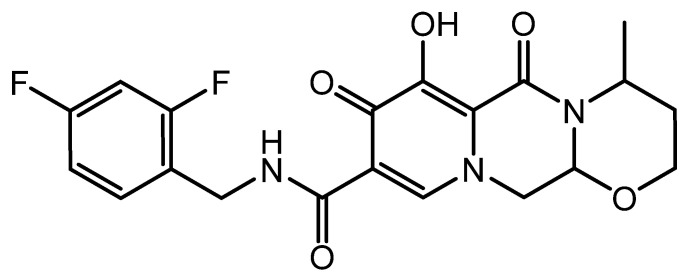
Chemical structure of darunavir.

Darunavir is a nonpeptidic protease inhibitor (Figure 1), which has been demonstrated to be effective against wild-type HIV. It has become an integral part of antiretroviral therapy, which is widely regarded as the key advancement in the area of HIV treatment [6]. The molecular weight of darunavir is 593.73 g/mole, and it is highly lipophilic (log *p* 3.94), with limited aqueous solubility (0.15 mg/mL at 20 °C) [7]. The therapeutic efficacy and safety of darunavir, when used in combination with other antiretroviral medications, have been proven through several clinical investigations [8,9].

Intranasal drug delivery has become an emerging alternative for the local and systemic therapy of different pharmaceutical actives [10]. The intranasal route of administering drugs is a noninvasive method for delivering antiretroviral medicines into the brain and CNS, bypassing the blood–brain barrier. The olfactory and trigeminal nerves offer unique connections between the brain and the external environment, which, in turn, can transport medications directly from the nasal cavity to the brain via the intranasal pathway, thus bypassing the blood–brain barrier [11]. The intranasal route is divided into two sections, one intracellular, and the other extracellular. In the intracellular pathway, endocytosis occurs in olfactory sensory cells, which is accompanied by axonal transport to synaptic clefts in the olfactory bulb, where the drug is exocytosed. In the extracellular method, drugs are carried directly into the cerebral spinal fluid by first entering through the paracellular space over the nasal epithelium, and then through the perineural space to the brain’s subarachnoid space [12]. The intranasal delivery approach works well for medications that are unable to enter the blood–brain barrier and exhibit action in the CNS, eliminating the need for systemic distribution and reducing systemic side effects. Intranasal therapy can be easily administered by patients, which provides higher patient compliance, the quick onset of action, avoids first-pass metabolism, and can provide direct delivery to the brain [13]. Moreover, the mucus membrane is highly vascularized and possesses high permeability. Given the nasal cavity’s anatomical features, the nasal route could be an effective choice for delivering antiretroviral drugs straight from the nose to the brain. Indeed, it has been demonstrated that the nasal-mucosa-tight junctions can be reversibly unblocked, even for large molecular actives, including peptides and proteins [14]. This, in turn, leads to the delivery of various biologics, which is evidenced by the large number of commercial products available and many more clinical trials [15]. In addition, various categories of drugs, including those for AIDS, Parkinson’s, Alzheimer’s, and cancer, have been used for the brain-targeted delivery by using the nasal route [16]. A large number of antiretroviral therapeutic molecules from various categories have been approved by the United States Food and Drug Administration (US FDA) for the treatment of neuroAIDS [2]. Many formulation strategies have been assessed to deliver these therapeutics to provide direct nose-to-brain delivery [16,17]. Drops, sprays, gels, powders, inserts, ointments, and other nasal preparations are utilized to transport the medicine to the target site, the brain [13]. In situ gelling techniques are commonly used for brain targets among such preparations.

The development of controlled-release delivery systems for local and systemic medication has sparked a lot of interest in various in situ polymeric formulations. In situ gel-forming systems are an intriguing polymeric system that begins as a flowing water solution before being supplied, and then goes through a phase transition to make a viscoelastic gel in a physiological setting [13]. In situ gel-forming systems have a higher viscosity that transforms into a gel when triggered by different conditions [18]. Due to their ease of control and practical benefits, temperature-responsive hydrogels are perhaps the most frequently researched class of environment-responsive polymer systems in dosage-forms research [13,19]. Temperature change causes gelation in thermosensitive hydrogels, which are liquid at room temperature, but gel when in contact with bodily fluids. In reality, in situ sol–gel transition happens when thermosensitive polymers in the formulation come into contact with the nasal membrane’s physiological temperature. Poloxamers are synthetic triblock copolymers, which possess thermosensitive gelling capabilities, great aqueous solubility, less toxicity/irritation, and superior drug-release qualities, making them ideal for in situ gel preparations [20]. The thermoreversible property of Poloxamer is primarily because of its negative coefficient of solubility in block copolymer micelles. Typically, Poloxamer forms monomolecular micelles or multimolecular aggregates, depending on the concentrations, which results in gel formation [21]. The gelation temperature and gelation time are the critical factors to be estimated to optimize thermosensitive gel. The gelation temperature is described as the lowest temperature upon which the prepared formulation undergoes a phase change to improve viscosity or become a semisolid gel. In general, an excellent thermoresponsive in situ gel has a glass transition temperature over the room temperature (25 °C) and achieves gel–sol transition at the physiological temperature of the nasal cavity. Hence, it is preferred that nasal in situ formulations have a gelation temperature above 25 and below 34 °C. The use of Poloxamer alone generally does not provide enough mechanical strength and mucoadhesion [22]. Hence, mucoadhesive polymers are generally included in nasal in situ gel formulations to provide mucoadhesion and thereby offer greater residence in the application site. Carbopol is most widely used as a mucoadhesive agent in the in situ gel formulations due to its excellent mucoadhesive property, as well as the fact that it ensures good viscosity [23]. In light of this, the current study attempted to formulate a mucoadhesive darunavir-loaded thermosensitive gel for intranasal delivery that might provide appropriate adhesion, a longer residence time in the nasal mucosa, and prolonged drug release. Indeed, this is a first-of-its-kind study in terms of the use of a darunavir-loaded in situ gel for intranasal delivery. Preliminary formulations (F1–F9) were prepared and evaluated for various pharmaceutical properties. A 3^2^ factorial design was used to optimize and evaluate the in situ mucoadhesive nasal gel containing darunavir (0.2% w/v). The concentrations of Carbopol 934P and Poloxamer 407 were selected as influencing variables, and the gelation temperature and percentage of drug release were selected as a response. The optimized formulation was evaluated for drug interaction, thermal behavior, permeation, ciliotoxicity, and in vivo brain bioavailability in a rat model.

## 2. Results and Discussion

### 2.1. Solubility Determination

**Figure 2:**
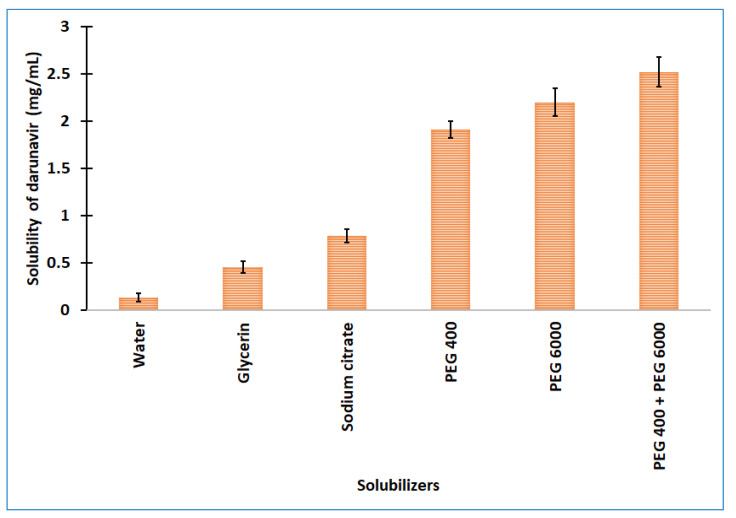
Solubility of darunavir in various solubilizers determined. The data presented are the average ± SD (*n* = 6). PEG: polyethylene glycol.

One of the most critical characteristics for attaining the correct drug level in systemic circulation, and for achieving the desired therapeutic response, is solubility. Poor water solubility is one of the major challenges that is faced by formulation scientists during the preformulation studies of new chemical entities and generic product development [24]. The experimentally determined aqueous solubility of darunavir was found to be relatively low (0.14 ± 0.05 mg/mL) to formulate it into a clear in situ gel. Therefore, different categories of solubilizers, such as cosolvents (glycerine and PEG 400), hydrotropes (sodium citrate), and water-soluble solid (PEG 6000), were evaluated for the solubility enhancement of darunavir. The solubilizers were selected on the basis of their renowned characteristics to improve the solubility of poorly aqueous-soluble drugs [25]. The observed solubility of darunavir in various solubilizers is represented in Figure 2. It is obvious from Figure 2 that the solubility decreases in the order: glycerin > sodium citrate > PEG 400 > PEG 6000 among the solubilizers tried. The highest solubility enhancement was observed with PEG 6000 (8.10 folds) and PEG 400 (7.05 folds). The higher solubility exhibited by PEGs could be due to the extensive hydrophilic interactions, as described in the literature [26]. Though PEG 6000 showed greater solubility, the usage of 30% could lead to higher consistency (viscous). Hence, a combination of PEG 400 (15% w/v) and PEG 6000 (15% w/v) was used to check the mixed solubilizers’ effect on the darunavir solubility. The data in Figure 2 show that the combination does enhance the darunavir solubility and is moderately higher than the PEG 400 or PEG 6000 alone, and the overall solubility enhancement was 9.29 folds. This higher enhancement in solubility could be related to the synergistic effect, in addition to the additive effect, on the basis of the concept of mixed solvency [27]. On the basis of the higher solubility value and to have good consistency, the mixed solubilizers that were constituted of 15% w/v of PEG 400 and PEG 6000 each were used for the further formulation of in situ gel.

### 2.2. Preparation of Darunavir Gel

**Figure 3:**
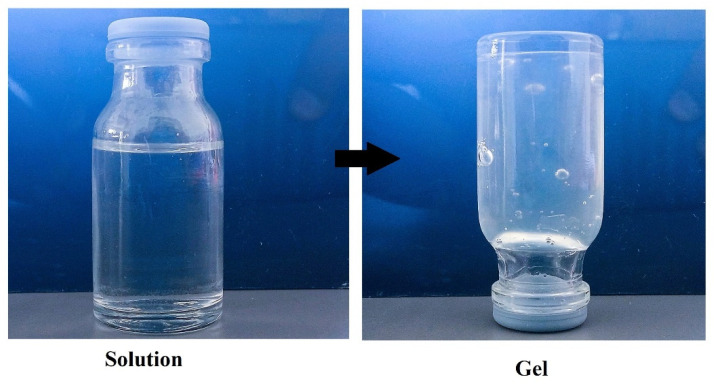
Representative photographs of nasal in situ formulation in solution and gel state.

The objective of developing in situ gel was to provide a patient-friendly droppable aqueous formulation for self-administration that forms an instant gel upon application on the nasal mucosa due to temperature change. Preliminary trials were carried out to assess the gelation temperature of the selected thermosensitive polymer (Poloxamer 407), in various concentrations, ranging from 15 to 25% w/v. It was noticed that, at low polymer concentrations (15–16% w/v), there was no phase transition until 40 °C. At 17% w/v, the viscosity increases at 38 ºC, but no phase transition took place until 40 °C. However, the increase in the polymer concentration from 18 to 25% w/v causes gelling, and, indeed, the gelation temperature decreases with an increase in the polymer content (Supplementary Table S1). The Poloxamer concentrations, which ranged between 18 and 22% w/v, showed a gelation temperature that was suitable for nasal application. Hence, these concentrations were included in the preliminary batches (F1–F9) of the nasal in situ gel containing darunavir (Table 1). The drug level in the preliminary batches was fixed at 0.2% w/v, on the basis of the solubility data, as this was the highest level of drug that could be incorporated to formulate it into a clear in situ gel. Similarly, the solubilizer concentration (15% w/v of PEG 400 and PEG 6000 each) was used in the formulation, as these mixed solubilizers had greater solubility. On the basis of the literature, three different concentrations (0.1, 0.3, and 0.5% w/v) of Carbopol were incorporated in the formulations as mucoadhesive polymers. Additionally, a renowned preservative (methylparaben at 0.05% w/v) was also included in the final composition to prevent microbial growth. Representative photographs of prepared the in situ formulations in solution and gel state are depicted in Figure 3.

### 2.3. Evaluation of Preliminary Batches

#### 2.3.1. Appearance, pH, and Drug Content

The prepared formulations of nasal in situ gel (F1–F9) were evaluated for basic parameters, such as clarity, pH, and drug content, and the data are summarized in Table 2. Indeed, the visual inspection signifies that all formulations were clear and transparent and absent of turbidity, and free from any particulate matter. The literature suggests that the physiological pH of the nasal mucosa is 6.3; hence, formulations with a pH range from 4.5 to 6.5 can avoid nasal irritation [10]. The pH of prepared in situ gels has a range of 4.8 to 5.6, and it did not significantly vary among the formulations studied. Therefore, the prepared formulations are safe and could be used for nasal application. However, a general trend noticed is that the increase in the Poloxamer content moderately enhances the pH; in contrast, the increase in the Carbopol concentration slightly decreases the formulation pH, which is in agreement with earlier studies [28,29]. The drug content is an important quality-control parameter that assesses the drug level in formulations and is evaluated during the formulation development [30]. The drug content observed in the prepared formulations indicates adequate darunavir content (>97%) in all gels and was comparable (Table 2). The higher and comparable drug level in the formulations also signifies that the formulation composition did not influence the content uniformity.

#### 2.3.2. Gelation Temperature

The gelation temperature plays a crucial role in nasal in situ formulations. When liquid solution is converted into gel phase, triggered by any stimuli, such as temperature, it is called the gelation temperature. It has been described that the gelation temperature of nasal in situ formulations should be more than 25 °C, which could overcome various issues that pertain to the production, storage, as well as the administration, of in situ gels [31]. However, if the gelation temperature of gel is higher than 35 °C, then other issues, such as the handling and administration of the product, may occur, and the gel may drain out of the nasal cavity. As the nasal temperature is 34 °C, the ideal temperature for the nasal application of in situ gel could be in the range of 28–32 °C [32]. Thus, the formulation development needs to take account of this parameter more seriously. The literature reveals that the gelation temperature of Poloxamer could be influenced by the inclusion of other ingredients in the gels [20,21,32]. A comparison of the data from Supplementary Table S1 (pure Poloxamer solution) and Table 2 (gels) indicates that the gelation temperature of the prepared gels decreased by the formulation components. The addition of solubilizers (PEG 400 and PEG 6000), as well as the mucoadhesive polymer (Carbopol), could influence the gelation temperature of thermoreversible gel, as demonstrated in the literature [20,21,32]. The observed gelation temperature of the prepared formulations has a range of 22–36 °C. Indeed, a significant difference (*p* < 0.05) in the gelation temperature was noticed with the increase in the Carbopol content (0.1–0.3% w/v) in the formulations studied. Hence, this parameter was considered as a response variable in the optimization study. The results also indicate that the formulations with 20% Poloxamer (F3–F5) showed gelation temperatures (28–32 °C) suitable for nasal application in terms of the gelation temperature.

#### 2.3.3. Mucoadhesive Strength

The adhesion of gel with the nasal mucus membrane is essential to enhance the residence time at the site of application and thereby improve the therapeutic effect. In general, the mucoadhesive strength is measured to evaluate the retention capacity of developed nasal in situ gels [33]. Depending on the mucoadhesive strength, it can prevent drainage from the nasal cavity and also in the nasopharynx. The higher the strength of the mucoadhesion, the more retention will be in the nasal mucosa [34]. The adhesion of gel to the nasal mucosa would potentially enhance the permeation rate of the drug through mucosal epithelial layers, and thereby achieve an effective concentration at the target site of the brain. Various polymers from different categories have been used as mucoadhesive agents in nasal in situ gels. Among them, the Carbopols have been widely used, as they exhibit excellent mucoadhesion strengths at low concentrations and are capable of prolonging the drug release [35]. The Carbopol 934 polymer that was used in the current study is a polyacrylate polymer with numerous carboxylic groups that is capable of forming hydrogen bonds with nasal mucus membranes, resulting in increased mucoadhesive strength [21]. The results in Table 2 indicate that the mucoadhesive strength of prepared formulations has a range from 31 to 58 mN, which is consistent with the reported results [35,36,37]. It is also evident that the increase in the Carbopol content (0.1–0.5% w/v) proportionally increases the mucoadhesive strength and was found to be statistically significant (*p* < 0.05). However, the values observed with all of the formulations here indicate adequate mucoadhesion and may prolong the retention of the prepared gels, which, in turn, increases the permeation of the drug.

#### 2.3.4. Viscosity and Spreadability

The rheological properties, such as viscosity and spreadability, are interrelated and play an influential role in the formulation of in situ gels, as well as in their efficacy. Adequate viscosity is necessary to extend the nasal residence time. For all formulations, the viscosity was found in the range of 3400–8000 cPs at 30 °C (Table 2). The results observed indicate that the increase in the quantity of the thermoresponsive polymer (Poloxamer) or mucoadhesive polymer (Carbopol) increased the viscosity of the in situ gel. However, the increase in viscosity was more prominent with Carbopol and increased in a concentration-dependent manner, as compared to Poloxamer (Table 3), which is also consistent with the earlier studies [20]. Spreadability is also an important parameter for the in situ gel to have ease of application and ease of spreading on nasal mucosa without leakage after administration. It was observed that the spreadability of formulated gels was in the range from 13.19 to 21.17 cm^2^/min. Indeed, the observed data in all of the tested in situ gel formulations have adequate spreadability and could be suitable for nasal application, according to the earlier studies [38].

#### 2.3.5. Drug Release

The release of drugs from in situ gel formulations is critical for absorption and therapeutic response. Thus, in situ nasal gel formulations must be carefully designed to prolong the duration of residence in the nasal cavity to achieve the desired drug release over the nasal mucosa [21]. Nasal in situ gels (F1–F9) were subjected to in vitro drug release for 8 h, and the cumulative amount of the drug released is presented in Supplementary Figure S1 and Table 2. The study results signify that the release of darunavir (86–99%) from prepared in situ gels was significantly influenced by the concentrations of the Carbopol polymer. The increase in the concentration of mucoadhesive polymer decreased the release rate of darunavir from in situ gels. For instance, the cumulative amount of drug release at 4 h with 0.1% Carbopol (68–79%) was relatively higher, as compared to 0.3% (56–65%) and 0.5% (52–56%) of Carbopol in the formulations. These findings suggest that, when the concentration of Carbopol increases, the gel’s structure served as a resistive barrier to drug release. Hence, the possible clarification for the increased resistance could be due to a reduction in the number and size of water channels inside the gel structure, as well as an increase in the density of the three-dimensional network, as described in the literature [39].

### 2.4. Optimization of Variables Using 3^2^ Factorial Designs

Prepared design batches (D1–D9) demonstrated various pharmaceutical properties well within acceptable limits and showed favorable rheological properties with adequate mucoadhesion. The gelation temperature and the % drug release in 8 h for the design batches were taken as dependent variables, and the experimental values are recorded in Table 3. The polynomial quadratic equation was used to evaluate the results of both response variables. The equations derived for both responses (Y_1_ and Y_2_ for a full and reduced model, respectively) were transformed and investigated. In general, the values of the coefficients and mathematical signs carried by polynomial equations can be used to make conclusions (i.e., negative or positive).

#### 2.4.1. Gelation Temperature (Y_1_)

From the data, the polynomial equation of the full model for the response (Y_1_) (i.e., the gelation temperature) was derived as shown below:

Y_1_ = 30.34 + 1.12X_1_ − 1.95X_2_ − 0.20X_1_X_2_ − 0.0167X_1_^2^ + 0.2833X_2_^2^

The value of r^2^ was found to be 0.9988. From the equation, it was observed that the positive value of the coefficient of X_1_ indicates a similar proportionality effect of X_1_, and the negative value of the coefficient of X_2_ indicates the opposite effect of X_2_ on the gelation temperature. The Poloxamer concentration from 19 to 21% w/v causes gelling, and, indeed, the gelation temperature decreases with an increase in the polymer content. From the *p*-value, it was observed that the effects of X_1_ and X_2_ are more significant. So, the polynomial equation was converted into reduced form as (*p* < 0.05).

Y_1_ = 30.34 + 1.12X_1_ − 1.95X_2_ − 0.20X_1_X_2_ + 0.2833X_2_^2^

**Figure 4:**
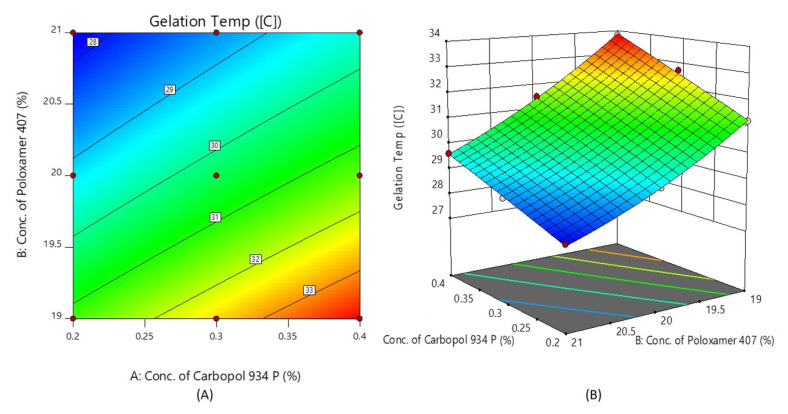
Contour plot (**A**) and 3D surface plot (**B**) show the effects of the concentrations of Carbopol 934P and Poloxamer 407 on gelation temperature.

Figure 4 shows the response surface plot and contour plot of the concentration of Carbopol 934P (X_1_) and the concentration of Poloxamer 407 (X_2_) versus the gelation temperature. It may also be observed that, due to its temperature-triggered property, X_2_ appears to decrease the gelation temperature by increasing its concentration.

#### 2.4.2. % Drug Release after 8 h (Y_2_)

The polynomial equation for the % drug release after 8 h using the full model is derived below:

Y_2_ = 93.47 − 4.53X_1_ − 2.83X_2_ + 0.4250X_1_X_2_ − 1.20X_1_^2^ − 0.3000X_2_^2^

The value of r^2^ was found to be 0.9967. From the value and mathematical sign of the coefficient of X_1_ and X_2_, this indicates that X_1_ and X_2_ both have a negative effect on the % drug diffusion after 8 h. As the value of X_1_ and X_2_ increases, the % drug release decreases, and specifically for the concentration of Carbopol (X_1_). This is due to a reduction in the number of aqueous channels inside the gel, as well as an increase in the density of the three-dimensional network, as reported in the literature [39]. It was also shown from the *p*-value that, from all the factors, only X_1_, X_2_, and X_1_^2^ are significant. So, the polynomial equation has been reduced into its reduced form as:

Y_2_ = 93.47 − 4.53X_1_ − 2.83X_2_ − 1.20X_1_^2^

**Figure 5:**
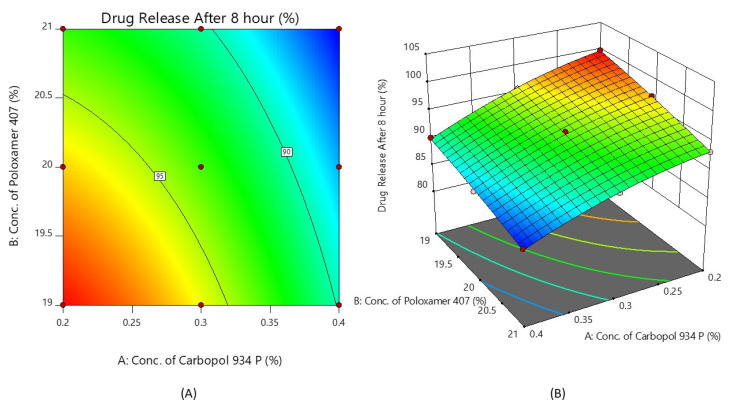
Contour plot (**A**) and 3D surface plot (**B**) show the effects of the concentrations of Carbopol 934P and Poloxamer 407 on drug release after 8 h.

Figure 5 shows the response surface plot and the contour plot of the concentration of Carbopol 934P (X_1_) and the concentration of Poloxamer 407 (X_2_) versus the % drug release after 8 h. It may also be observed that X_1_ and X_2_ both appear to affect the preparation of nasal in situ gel. It is possible to argue that the percentage of medication release can be altered by choosing the right X_1_ and X_2_ levels.

#### 2.4.3. Validation of Statistical Model

**Figure 6:**
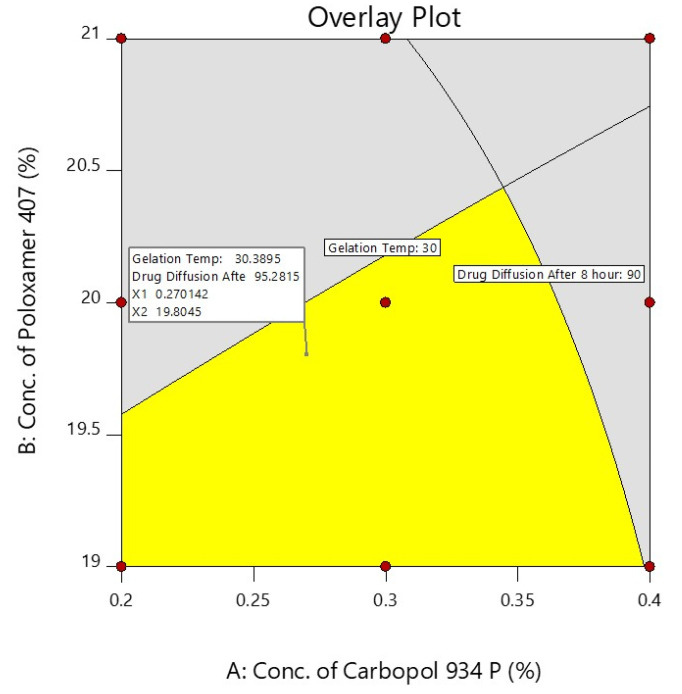
Overlay plot of independent factors and their responses.

A checkpoint batch was prepared to confirm the validity of the equation generated by regression analysis. The prepared checkpoint batch was evaluated for the gelation temperature and the % of darunavir release at 8 h, and these observed values were compared to the predicted values obtained from the overlay plot, as shown in Figure 6 and Table 4.

The differences between the predicted and experiential values were insignificant. The selected experimental model’s validity was confirmed by the good agreement between the predicted and experimental values. On the basis of the design and overlay plot studies, formulation D7 was selected as an optimized batch, which contains the middle value of both the variables (X_1_—0.3% and X_2_—20%), and it showed around a 30 °C gelation temperature and 93.93 (~94%) drug release after 8 h, which are in the well-accepted criteria.

### 2.5. Drug Release

**Figure 7:**
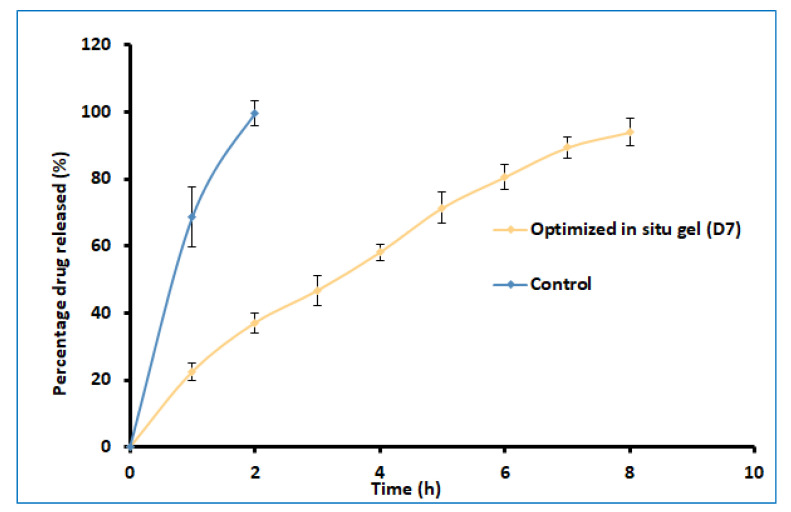
Comparison of percentages of darunavir release from optimized in situ gel (D7) and control (suspension). The data presented are the average ± SD (*n* = 6).

The delivery of drugs from gels is critical for absorption and the resultant therapeutic response. Figure 7 shows a profile of the total amount (%) of darunavir released from D7 and the control. Two distinct release profiles were noticed for the in situ gel and the control. A steady and prolonged drug release was noticed in D7, confirming the feasibility of the developed gel to release the drug in a steady period of 8 h. In contrast, the release was rapid and complete (2 h) in the control formulation. The kinetics of the drug-release data were analyzed for zero-order, first-order, the Higuchi model, and the Korsmeyer–Peppas model. The Korsmeyer–Peppas model has a higher r^2^ value (0.997), a lower sum of squares of the residuals value (20.47), and a lower Fischer Ratio value (2.92). Hence, the optimized in situ gel formulation followed Korsmeyer–Peppas release kinetics, wherein it was found that the *n* value (0.7001) was greater than 0.45 (Supplementary Table S2), which indicates that the drug-release mechanism is anomalous [40].

### 2.6. FTIR

**Figure 8:**
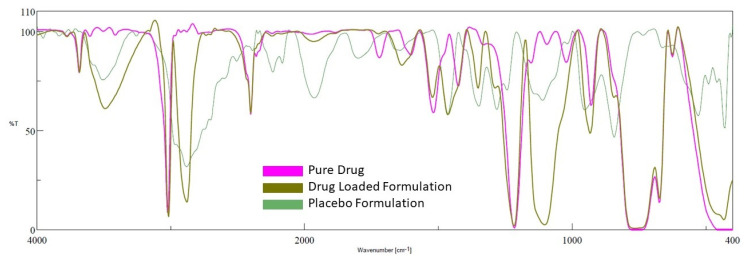
FTIR spectra of darunavir, placebo, and in situ gel formulation (D7).

FTIR is used in the preformulation investigations to examine the possible incompatibility of drugs with excipients, as well as to check the stability of the drug in the final gel formulation [41]. The excipients in the formulation may interact with active medicinal substances, causing molecular transformation, and, as a result, affecting the product’s stability [42]. The spectra of the drug, placebo gel, and selected formulation (D7) are presented in Figure 8. Pure darunavir showed absorption bands due to -NH_2_ (3683.37 cm^−1^), -C-H stretching (3023.84 cm^−1^), -N-H stretching (2402.87 cm^−1^), -C=O stretching (1720.19 cm^−1^), -C=C stretching (1600.63 cm^−1^), -C-N bending (1519.63 cm^−1^), -C=H bending (1427.07 cm^−1^), -C-O stretching (1214.93, 1095.37, 1022.09 cm^−1^), and -HC=CH- bending (929.521 cm^−1^). As shown in Figure 8, in the spectra of the placebo formulation, the main peaks of the polymers are visible. The FTIR spectra of the in situ gel formulation reveal all of the drug and polymer characteristic peaks, with minimal shift because of overlapping excipient peaks. The results confirm the absence of incompatibility between the drug and the other excipients used in the in situ gel formulation.

### 2.7. DSC Analysis

**Figure 9:**
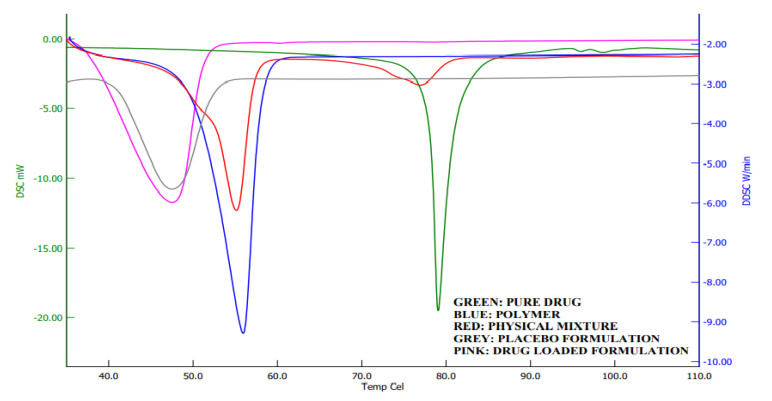
Differential scanning calorimetric curves of darunavir, Poloxamer, physical mixture, placebo, and in situ gel formulation (D7).

Calorimetry is a method for detecting the thermal behavior of formulation ingredients [30]. The DSC thermogram showed a sharp endothermic peak at 80 °C and 58 °C, which shows the melting points of darunavir [43] and Poloxamer [44], respectively. The DSC of the physical mixture showed two peaks, but with reduced intensity compared to the pure drug and polymer, which may be due to the dilution of the drug with the polymer. However, there was no interaction of the drug and polymer noticed. In thermograms of in situ gel (Figure 9), a single endothermic peak around 45 °C was noticed, which is also noticed in the placebo formulation. This is probably the shift in the Poloxamer melting point that is due to the formation of a gel. No peak of the drug is seen in the formulation around 80 °C, which indicates that there is complete solubilization of the drug inside the in situ gels.

### 2.8. Ex Vivo Permeation Studies

**Figure 10:**
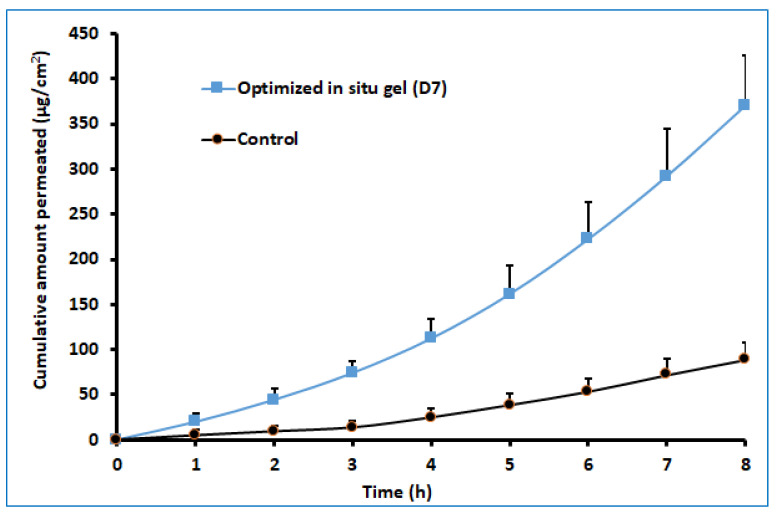
Comparison of ex vivo darunavir permeation using isolated sheep nasal mucosa from optimized in situ gel and control (suspension). The data presented are the average ± SD (*n* = 6).

The ex vivo permeation study provides significant insight into the formulation behavior under the in vivo conditions [45]. It is well known that the properties of the therapeutic molecules, as well as the physiology of the biological barriers, regulate drug transport through any membrane. The darunavir permeation from the optimized in situ gel and the control (suspension) was evaluated ex vivo by using sheep nasal mucosa, which has more resemblance to human nasal mucosa [46,47]. The observed permeation of both formulations is depicted in Figure 10. It is apparent from Figure 10 that the drug transport from the in situ gel is significantly higher (*p* < 0.005) than in the control. The greater drug permeation (cumulative amount permeated in 8 h was ~370.17 µg/cm^2^) noticed with the gel formulation could be related to the higher drug solubility. Being a BCS class II drug, an increase in the solubility of this drug is likely to provide higher permeability across the nasal membrane. A higher steady-state flux (~49.83 µg/cm^2^/h) and permeability coefficient (1.25 × 10^−2^ cm/h) were observed with the gel formulation in comparison to the control (flux: ~49.83 µg/cm^2^/h, and permeability coefficient: ~3.08 × 10^−3^). However, the lag time for both formulations was comparable (1.25 and 1.39 h for in situ gel and control, respectively). The results of this study signify that the optimized in situ gel formulation could potentially augment the nasal delivery of darunavir.

### 2.9. Nasal Ciliotoxicity Study

**Figure 11:**
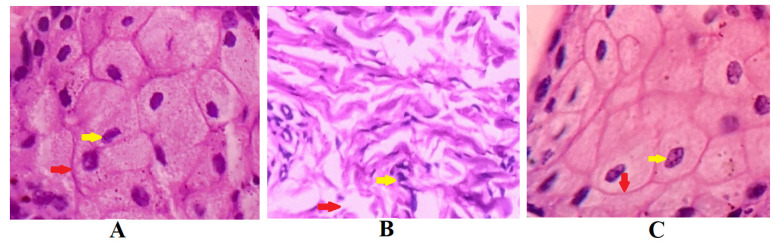
Histopathological examination of sections of sheep nasal mucosal membrane. (**A**) Negative control: hematoxylin and eosin staining of nasal mucosa membrane applied with phosphate buffer reveal unaffected nasal cells with intact basement membranes (red arrows), as well as undamaged glandular cells with clear nuclei (yellow arrow). (**B**) Positive control: nasal mucosa treated with isopropyl alcohol suffered severe cellular and histological damage. The treatment with isopropyl alcohol disrupted basement membrane (yellow arrow) and loss of nucleus of glandular cells to form a multinucleated cells. (**C**) Test: nasal in situ gel of darunavir when exposed to nasal tissues of the sheep showed intact basement membrane (red arrow) and normal arrangement of the glandular cells with well-defined nuclei (yellow arrow), indicating no signs of toxicity of the formulation.

Figure 11 demonstrates the results of the nasal ciliotoxicity studies conducted after treatment with the in situ gel of darunavir (test), phosphate buffer (negative control), and isopropyl alcohol (IPA) (positive control). An examination of the stained sheep nasal mucosa was performed by using a light microscope. The histopathological examinations of the nasal mucosa applied with the negative control (Figure 11A) and the in situ gel of darunavir (Figure 11C) demonstrated no cellular damage or any kind of toxicity of the nasal mucosa, indicating the potential of the in situ gel of darunavir to preserve the cellular integrity. However, the treatment with IPA showed significant damage to the cellular morphology.

### 2.10. In Vivo Pharmacokinetic Studies

The key objective of the study was to enhance the brain bioavailability of darunavir by developing a gel formulation for nasal application. Due to the existence of a highly selective blood–brain barrier, the drug concentration–time profile in the brain may be considerably different than that in the blood [48]. Drugs within the brain undergo various distribution and elimination processes, such as diffusion, the bulk flow of the brain extracellular fluid and cerebrospinal fluid, extracellular–intracellular exchange, specific binding to the target site, and nonspecific binding to brain-tissue components and metabolism [49]. Establishing a quantitative relationship between the spatial drug-distribution processes within the brain and the concentration of the drug at the target site of the brain is important to predict the desired clinical effect. Both structural characteristics of the brain and drug properties can influence the distribution of drugs inside the brain. Mathematical modeling describing drug transport through the brain capillary system, drug transport across the blood–brain barrier, intra–extracellular exchange, drug binding within the brain, and drug metabolism within the brain could accurately predict the effect of a drug that targets the brain [49].

**Figure 12:**
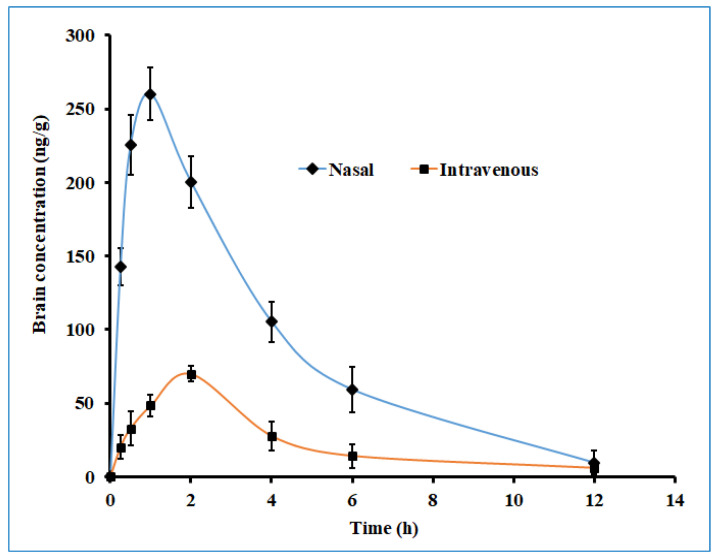
The brain drug concentration–time profile of darunavir after administration by nasal (optimized in situ gel, 30 µL, 2.4 mg/kg) and intravenous (solution, 0.4 mL, 2.4 mg/kg) routes in rats. The data presented are the average of six animals at every time point.

The brain drug concentration–time profile of darunavir following the single-dose intranasal administration of the optimized in situ gel and the intravenous administration of the solution in rats is presented in Figure 12, and the observed pharmacokinetic parameters are summarized in Table 5. Two distinct profiles were noticed for both nasal and intravenous routes of administration, with significantly higher (*p* < 0.005) drug levels being noticed with nasal delivery. Absorption through the nasal route seems to be very rapid, with a greater amount of darunavir reaching the brain within 0.25 h (~140 ng/g), as compared to intravenous delivery (~20 ng/g). Intranasal administration provided a lower T_max_ value (1 h) than its intravenous counterpart (2 h) (Table 5), suggesting the quick transport of darunavir through the nose to the brain. Intranasal administration showed a ~4-fold increase in the C_max_ value in the nasal route, as compared to intravenous therapy (Table 5). The higher C_max_ values noticed in the nasal route could again be attributed to the greater permeation of darunavir from the formulated in situ gels. The superior values in all of the pharmacokinetic parameters of interest noticed in the nasal administration of darunavir signify the potential of the optimized in situ gel to provide intimate contact with the mucus membrane and to be retained in the nostril for a prolonged period, and to effectively deliver the therapeutic. Followed by the C_max_, the brain drug levels in both therapies seem to decline rapidly, suggesting the quick clearance of the drug from the brain. The bioavailability of darunavir in intranasal delivery seems to be much higher, as evidenced by a greater AUC_0-α_ value (~3.5 folds higher than the intravenous route), which points to the higher absorption of the drug from the in situ gel. The low amount of darunavir that reaches the brain in intravenous therapy could be due to the poor transport of the drug through the blood–brain barrier, which is additionally protected by the tight junctions [5]. A relative comparison of the bioavailability of darunavir, quantified through AUC, showed a 3-fold higher concentration in the brain (Table 5) than in plasma, thus confirming the potential capacity and practical feasibility of intranasal gel for CNS delivery. Overall, the pharmacokinetic data here signify that the intranasal administration of the developed in situ gel showed significant improvement in the brain bioavailability of darunavir compared to intravenous administration.

**Figure 13:**
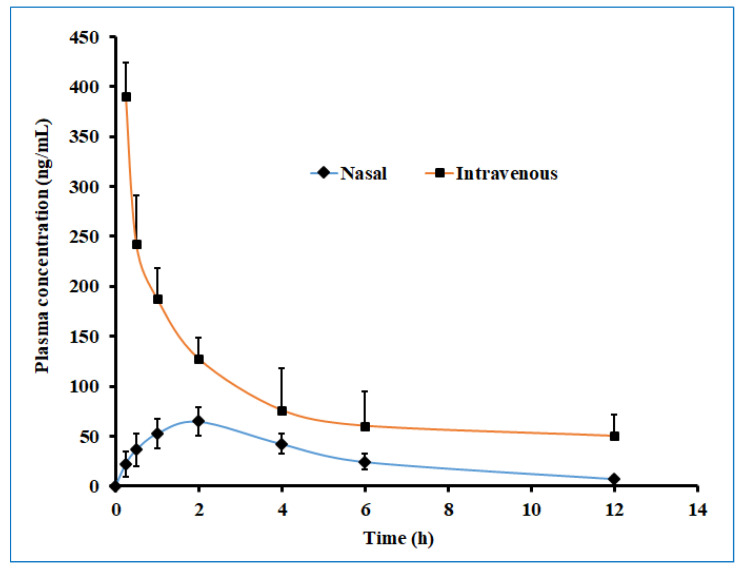
The plasma drug concentration–time profile of darunavir after administration by nasal (optimized in situ gel, 30 µL, 2.4 mg/kg) and intravenous (solution, 0.4 mL, 2.4 mg/kg) routes in rats. The data presented are the average of six animals at every time point.

Figure 13 displayed the plasma drug concentration–time profile of darunavir after administration by nasal and intravenous routes. It is apparent from Figure 13 that a high drug level in the plasma was yielded with the intravenous route as compared to intranasal delivery. Nevertheless, the calculated AUC_0-α_ in the intravenous route was ~3 fold larger than the AUC_0-α_ obtained for the intranasal route. The low drug plasma concentration–time profile observed with intranasal delivery suggested the low systematic exposure of darunavir through the paracellular transport mechanism. Lower C_max_ and AUC_0-α_ values were noticed with the nasal application (compared to a similar dose of darunavir given after intravenous administration (Table 5)), signifying the low absorption of the drug into the central compartment.

### 2.11. Stability Study

The physical and chemical stability of the optimized in situ gel was evaluated at two different conditions of temperature and humidity. It was observed that the formulation showed an insignificant difference in the parameters evaluated. There was no change in the appearance of the formulation under all storage conditions over 3 months. The drug content of the product was maintained throughout the study period. Similarly, the gelation temperature, mucoadhesive strength, viscosity, and drug release did not vary significantly and were comparable with the values observed at the initiation of the experiment. Thus, it can be concluded that the developed in situ gel formulation possesses sufficient stability for 3 months.

## 3. Conclusions

This study assessed the potential of developing an in situ gel system for the intranasal delivery of darunavir to the brain for treating neuroAIDS. The preliminary study was carried out to select the composition of various embodiments, as well as to assess the effect of polymers on the various physicomechanical properties of the gel. These trials helped to identify that the polymers (Poloxamer 407 and Carbopol 934P) influence the gelation temperature and drug release, and, hence, they were selected as the response variables. Then, a full factorial statistical design was performed to optimize the polymer concentration in the formulations (D1-D9) by considering both response variables. Indeed, the results of the optimization study indicate that both independent variables influence the response variable considerably. Optimized mucoadhesive in situ gel (D7) exhibited MH7, exhibited all physicochemical properties within satisfactory limits, and was found stable for three months. The developed thermoreversible in situ gels have a long residence time and they slowly released the drug over 8 h. The FTIR and DSC results confirm the absence of incompatibility between the drug and the other excipients used. The results of the permeation data indicate greater flux and permeability by the optimized mucoadhesive gel. The nasal ciliotoxicity data confirm the nontoxicity of the formulation. The in vivo study further substantiates the ex vivo data, as demonstrated by the higher drug level in the brain when delivered through the nasal route. Moreover, the systemic exposure to drugs from intranasal delivery was very low. A stability study signifies that the developed in situ gel formulation possess sufficient physical and chemical stability for three months. In a nutshell, the developed in situ mucoadhesive gel formulation proved to be successful for the nasal application of darunavir, and it could be an effective way of delivering this drug directly from the nose to the brain.

## 4. Materials and Methods

### 4.1. Materials

Darunavir and Poloxamer 407 were a gift sample from Emcure, Gandhinagar, India. Polyethylene glycol (PEG) 400, PEG 6000, glycerol, and sodium citrate were donated by Suvidhinath Laboratories, Baroda, India. Carbopol 934 P, methylparaben, and propylparaben were purchased from Chemdyes Corporation, Rajkot, India. Acetonitrile was purchased from Merck, Mumbai, India.

### 4.2. Analysis of Darunavir

The analysis of darunavir was performed by a high-performance liquid chromatography (HPLC) system (Shimadzu, Tokyo, Japan). A solvent system consisting of a combination of potassium phosphate buffer (10 mmol/L, pH ~ 3) and acetonitrile in 51:49 was used for the separation of darunavir using a monolithic C18 column (Zorbax, 150 mm × 4.6 mm, i.d, 5 µm) [50]. The solvents were adjusted to run at 0.6 mL/min across the column, and drug elution was monitored with a UV detector set to 202 nm. Nevirapine was used as an internal standard. The injection volume was 50 µL, and the retention time was seen at 3.6 min. Validation of the analytical method was conducted by performing various parameters, such as sensitivity, selectivity, linearity, accuracy, precision, ruggedness, and limit of quantification. The developed method showed linearity in the range of a 4–600 ng/mL concentration, with the value of r^2^ = 0.9978.

### 4.3. Solubility Determination

The saturation solubility of darunavir in water and different solubilizers was determined by an established method, according to the literature [51], at room temperature (25 ± 1 °C). Solubilizers (glycerin, sodium citrate, PEG 400, and PEG 6000), at a concentration of 30% w/v, were used to check darunavir solubility. In addition, a combination of PEG 400 and PEG 6000, at a concentration of 15% w/v, was also assessed. In brief, an excess amount of darunavir was added to prepare a saturated solution of darunavir in water and different solubilizers, and the vials were tightly closed. Vortex mixture was used to mix all the solutions for 10–15 min, and was then sonicated for 10 min. The solution mixtures were agitated mechanically for 12 h, kept aside for an hour to achieve equilibrium, and later filtered through a 0.22 µm membrane filter unit (Millipore Corporation, Bedford, MA, USA). Each sample was filtered and diluted using the mobile phase and analyzed using HPLC.

### 4.4. Preparation of Darunavir Gel

The gel was prepared using a process that has been previously reported [31]. The composition of prepared in situ gels (F1–F9) is summarized in Table 1. In brief, the required amount of Poloxamer 407 (18–22% w/v) was dissolved in a glass beaker containing cold distilled water and was stored at 4 °C to dissolve it completely. Carbopol 934P (0.1–0.5% w/v) was solubilized separately in water by keeping it for 24 h, and was slowly added to the above Poloxamer solution with constant stirring. The prepared gelling solution was added with darunavir (0.2% w/v), solubilizer (30% w/v), and methylparaben (0.05%). The preparation was mixed under constant stirring by a magnetic stirrer to make a homogeneous solution.

### 4.5. Evaluation of Prepared Gels

#### 4.5.1. Appearance

The formulation was evaluated for appearance by visual inspection on the basis of its clarity under sufficient lighting, and it was visualized against a black/whiteboard by inverting the sample to provide swirling action to check the motion of particulate matter and fibers. Moreover, it was observed for the presence of any particulate matter and the development of turbidity in the formulations [40].

#### 4.5.2. pH and Drug Content

The pH of prepared in situ gel formulations was determined by a pH meter (Mettler Toledo MP-220, Greifensee, Switzerland), which was previously calibrated. Determination of drug content was carried out by taking 1 g of in situ gel and mixing (5 min) with mobile phase using a laboratory mixer (EIE 405, EIE Instruments, Ahmedabad, India). The solution was further filtered (0.22 µm pore size) and the drug content was analyzed.

#### 4.5.3. Determination of Gelation Temperature

The gelation temperature of prepared gels was measured by the visual examination method mentioned in the literature [20]. Briefly, 5 mL of the formulation was taken in a 10 mL glass vial containing a magnetic bead and sealed. The vials were immersed in a water bath controlled with a thermostat, and the bath temperature was gradually increased from 20 °C to 40 °C by precisely measuring the temperature using a thermometer with a 0.1 °C scale range. The magnetic bead was stirred at a speed of 80 rpm. The temperature wherein the bead ceased rotating was used to determine the gelation temperature.

#### 4.5.4. Mucoadhesive Strength Determination

The force required to separate the in situ gel from sheep nasal mucosa was measured using a texture analyzer (Stable Microsystems, Surrey, UK). The testing was conducted on the freshly cut piece (2 cm × 2 cm) of isolated sheep nasal mucosa, previously equilibrated with simulated nasal fluid (comprised of sodium chloride: 8.77 g/L; potassium chloride: 2.98 g/L; calcium chloride: 0.59 g/L, adjusted to pH 6.5 using 0.1 M sodium hydroxide) for 15 min at 37 ± 0.5 °C. A portion of the nasal mucosa was glued to the top platen assembly, such that the mucosal side was facing outwards [37]. On the lower platen, a set amount of gel (500 mg) was applied. The upper probe was then let down at a pace of 0.5 mm/s onto the bottom platen until it made contact with a specified compressive force of 1 N and a contact time of 60 s. The probe was then detached at a distance of 15 mm at a speed of 0.5 mm/s [52]. The required force to separate the formulation from the sheep mucosa was determined.

#### 4.5.5. Viscosity

The measurement of viscosity of prepared gels was carried out using a Brookfield Viscometer (LVDVI prime, Middleborough, MA, USA) at a temperature of 30 ± 1 °C and an angular velocity of 20 rpm.

#### 4.5.6. Spreadability

Spreadability is calculated by the area covered by gel formulation per unit time (cm^2^/min). It was measured by using a 1 mL graduated pipette attached to the rubber bulb, and the pipette was fixed on the clamp vertically in such a way that there was a distance of 2 cm between the tip of the pipette and the horizontal surface of the Whatman filter paper (0.45 µm). Place 0.1 mL of formulation from the graduated pipette at the center of the filter paper. Spreadability was measured by calculating the surface area covered by formulation on filter paper at a fixed time interval of 20 s [38].

### 4.6. In Vitro Drug Release

Franz diffusion cell (Orchid Scientific and Innovative India Ltd., Nashik, India) was used for in vitro release study, with an effective surface area of 1.3 cm^2^. Drug release was carried out using simulated nasal fluid (pH 6.4) with the help of a cellophane membrane (MWCO 12–14 kDa) [53]. Briefly, gel equivalent to 4 mg of darunavir was applied on the surface of the previously soaked dialyzing membrane, which was kept between the receptor and donor compartment. The receptor compartment contains simulated nasal fluid (pH 6.4, 20 mL). The whole assembly of the diffusion cell was placed on a water bath, which was thermostatically controlled at 37 ± 0.5 °C, and the receiver solution was stirred at 50 rpm [54]. Withdrawal of samples was carried out at a periodic time up to 8 h and replaced with an equal volume of simulated nasal fluid (pH 6.4). The samples were diluted subsequently, and analytical estimation was performed for darunavir by HPLC. To pick the best-fit release model, various mathematical models, such as zero-order, Higuchi, Korsmeyer, Peppas, and others, were utilized. From the kinetic release data, the correlation coefficient (*r*^2^), as well as release kinetics, were determined [55].

### 4.7. Optimization of Variables Using 3^2^ Factorial Designs

Generally, factorial designs were conducted to study the influence of several factors on experimental outcomes. On the basis of influencing factors, a full factorial design with 2 factors at 3 levels was selected for optimization. The concentrations of Carbopol 934P (X_1_) and Poloxamer 407 (X_2_) were selected as influencing variables, and gelation temperature (Y_1_) and % drug release (Y_2_) at 8 h were selected as the responses. As the factors are quantitative, it was required that the experiment be performed at three levels when it was expected to have curvature of the response [56]. The factors were studied at three levels (−1, 0, +1), demonstrating low, medium, and high, respectively, as shown in Table 6. Statistical models having polynomial equations and interaction terms were used to evaluate response. A total of nine experimental runs were carried out, and formulations were coded from D1 to D9. Prepared design batches were analyzed for clarity, pH, drug content, gelation temperature, mucoadhesive strength, viscosity, spreadability, and drug release after 8 h. Responses were analyzed by the statistical model.

### 4.8. Fourier Transform Infrared (FTIR) Spectroscopy

Spectra of pure darunavir, optimized formulation, and placebo gel were recorded to assess the possible interaction between drug and excipients. Samples (3–5 mg) were dissolved in 1 mL chloroform and 100 µL solution injected in the liquid cell of the IR compartment, and scanned between wavenumbers 4000 and 400 cm^−1^ using FTIR (6100, Jasco, Tokyo, Japan), and the spectra were compared for changes in drug peaks.

### 4.9. Differential Scanning Colorimetric (DSC) Analysis

DSC method was used to study the thermal behavior of drugs, Poloxamer, physical mixture, placebo, and in situ gel formulation using the Hitachi DSC instrument (DSC 7020, Japan). Weighed samples (5 mg) were placed in a separate aluminum crucible (25 μL), crimped, and nonhermetically sealed, while an empty pan was used as a standard. Thermograms were taken in an inert atmosphere by purging nitrogen gas at a flow rate of 50 mL/min, with a temperature range of 30–110 °C, at a uniform heating rate of 10 °C/min. Drug, polymer, and physical mixture were directly analyzed in solid form, whereas in situ gels of darunavir were first freeze-dried, and then analyzed for thermal analysis. The final formulation was kept for the primary freezing at −40 °C in the deep freezer (RQVD-300 PLUS; Remi, Mumbai, India) for 36 h. After that, final drying was carried out at −80 °C in the deep freezer (TFD 8503; ilShin Biobase, Dongducheon, Korea) for 12 h and used for DSC analysis.

### 4.10. Ex Vivo Permeation

Drug diffusion of prepared in situ gels was determined using the Franz diffusion cell setup described under in vitro release study [57]. The nasal cavity of sheep was collected from the nearby slaughterhouse, and fresh nasal mucosa was removed and stored in saline water at −20 °C in a deep freezer. Stored tissue was placed in a diffusion cell between two compartments. The mucosal surface was in touch with the formulation, while the receiver compartment contained simulated nasal fluid [58]. In situ gel containing darunavir (D7) equivalent to 4 mg or aqueous suspension (control) was applied to donor compartment. The diffusion cell was kept under agitation by a magnetic stirrer at 50 rpm, while the temperature of the system was set at 34 ± 0.5 °C by a circulating water bath. Samples were withdrawn every hour until 8 h, and an equal volume of samples of simulated nasal fluid (pH 6.4) was replaced.

### 4.11. Nasal Ciliotoxicity Studies

Nasal ciliotoxicity of optimized formulation (D7) was performed ex vivo on the nasal mucosa of sheep, according to the institutional ethical guidelines (Protocol No. IP/PCEU/FAC/27/2020/029). Histopathological studies were performed to evaluate nasal toxicity on tissue. Three identically sized sheep nasal mucosa pieces (A, B, and C) were chosen and put on Franz diffusion cells [35]. As a negative control, A was applied with 0.5 mL of phosphate buffer, B was applied with 0.5 mL of isopropyl alcohol as a positive control, and C was applied with 0.5 mL of optimized nasal in situ gel formulation of darunavir (D7) as a test. The mucosa was washed with nasal saline fluid after 6 h and subjected to histological tests utilizing hematoxylin–eosin staining [59]. A light microscope (ZEISS, Axioscope 5, Jena, Germany) with a magnification of 400 was used to examine the stained slides, and images were taken with a camera mounted to the microscope.

### 4.12. In Vivo Pharmacokinetic Studies

A preclinical study employing animals was conducted, according to the protocols of the Institutional Animal Ethical Review Board (Protocol No. IP/PCEU/FAC/27/2020/029). Each male Sprague Dawley rat, with an average weight of 250–300 g, was utilized for the estimation of various pharmacokinetic parameters. All animals were housed in separate cages in a well-controlled room (20 ± 2 °C and alternating 12 h light/12 h dark cycle) and were given free access to standard diet and water. Animals were fasted for at least 12 h before the administration of the assigned formulation. Rats were separated into Group I and Group II, with six animals at every time point. Animals in Group I received optimized in situ gel formulation with 0.2% w/v of darunavir (30 µL, 2.4 mg/kg) intranasally, while Group II rats were administered with drug solution (prepared using 5% dimethyl sulfoxide, 45% ethanol, and 40% normal saline to achieve 1.5 mg/mL darunavir solution) [60] intravenously (2.4 mg/kg) through the tail vein. Blood samples were withdrawn in heparin precoated tubes from the tail vein at specific time intervals (0.25, 0.5, 1, 2, 4, 6, and 12 h). Protein precipitation in plasma was carried out by adding an equal volume of acetonitrile [61] containing nevirapine (internal standard), and samples were subjected to centrifugation (1789× *g* for 15 min) and then filtered. Similarly, isolated brain tissues (collected by sacrificing rats after inducing anesthesia using intraperitoneal urethane (1.0 g/kg)) were homogenized in methanol and mixed with acetonitrile containing internal standard, and the solvent was filtered. Samples of plasma and brain extract were analyzed using HPLC. The mean recovery of drugs from plasma and brain were 97.68 and 94.22%, respectively. Various pharmacokinetic parameters, such as C_max_, T_max_, and AUC _0-t_, were estimated by noncompartmental analysis [62].

### 4.13. Stability Studies

The stability of the selected in situ gel was examined at temperatures of 25 °C ± 2 °C (60 ± 5% RH) and at 4 °C ± 2 °C (55 ± 5% RH) for 3 months, according to the ICH guidelines [56,63]. Samples were kept in glass vials covered with a screw cap. Every month, samples were taken and evaluated for appearance, pH, drug content, gelation temperature, mucoadhesive strength, and drug release.

### 4.14. Data Analysis

The outcomes of the experimental data were examined quantitatively using one-way ANOVA or the T-test in GraphPad Prism 6 (Graph-Pad Software, Inc., La Jolla, CA, USA). Statistical significance was defined as a difference in data with a *p*-value of less than 0.05.

## Figures and Tables

**Table 1:** Compositions of preliminary batches of nasal in situ gel of darunavir.

Composition	F1	F2	F3	F4	F5	F6	F7	F8	F9
Darunavir (%)	0.2	0.2	0.2	0.2	0.2	0.2	0.2	0.2	0.2
PEG 400 + PEG 6000 (%)	15 + 15	15 + 15	15 + 15	15 + 15	15 + 15	15 + 15	15 + 15	15 + 15	15 + 15
Poloxamer 407 (%)	18	18	18	20	20	20	22	22	22
Carbopol 934P (%)	0.1	0.3	0.5	0.1	0.3	0.5	0.1	0.3	0.5
Methyl paraben (%)	0.05	0.05	0.05	0.05	0.05	0.05	0.05	0.05	0.05
Distilled water (%)	Up to 100	Up to 100	Up to 100	Up to 100	Up to 100	Up to 100	Up to 100	Up to 100	Up to 100

**Table 2:** Physicochemical characteristics of preliminary batches (F1–F9) of nasal in situ gel containing darunavir.

Batch Code	Appearance	pH	% Drug Content	Gelation Temperature (°C)	Mucoadhesive Strength (mN)	Viscosity (cP)	Spreadability (cm^2^/min)	% Drug Release after 8 h
F1	Clear	5.19 ± 0.72	98.19 ± 2.74	36.66 ± 0.27	31.92 ± 3.36	3430 ± 790	21.17 ± 3.26	99.58 ± 1.26
F2	Clear	4.94 ± 0.10	99.34 ± 1.19	34.29 ± 0.51	44.95 ± 3.21	5855 ± 735	18.22± 2.27	96.85 ± 2.05
F3	Clear	4.81 ± 0.93	97.25 ± 2.36	33.34 ± 0.38	57.78 ± 4.05	8076 ± 852	14.03 ± 2.75	93.18 ± 2.79
F4	Clear	5.36 ± 0.46	98.73 ± 3.96	32.16± 0.19	33.20 ± 3.02	3891 ± 726	20.75 ± 2.88	99.02 ± 1.91
F5	Clear	5.12 ± 0.25	99.12 ± 1.16	30.28 ± 0.43	46.55 ± 2.34	6230 ± 928	17.64 ± 2.23	95.24 ± 3.37
F6	Clear	4.89 ± 0.65	98.38 ± 2.27	28.71 ± 0.30	58.06 ± 3.74	8666 ± 1082	13.62 ± 1.72	92.77 ± 4.22
F7	Clear	5.64 ± 0.67	98.39 ± 2.88	26.65 ± 0.48	33.70 ± 2.88	3979 ± 960	20.44 ± 2.38	98.29 ± 2.26
F8	Clear	5.27 ± 0.36	99.27 ± 1.45	23.85 ± 0.16	45.01 ± 3.06	6991 ± 854	16.92 ± 2.74	94.16 ± 3.26
F9	Clear	5.01 ± 0.86	98.71 ± 3.11	22.21 ± 0.62	57.96 ± 4.16	8011± 974	13.19 ± 1.11	91.33 ± 4.68

**Table 3:** Responses of 3^2^ full factorial design batches.

Batch	Response	
	Gelation Temperature (°C)	% Drug Release after 8 h
D1	29.2 ± 0.42	96.82 ± 3.62
D2	31.5 ± 0.27	89.34 ± 4.95
D3	31.2 ± 0.22	99.81 ± 1.06
D4	27.8 ± 0.30	93.25 ± 2.78
D5	33.8 ± 0.34	90.13 ± 4.39
D6	29.6 ± 0.44	88.22 ± 4.90
D7	30.3 ± 0.28	93.93 ± 3.11
D8	28.6 ± 0.35	90.24 ± 4.68
D9	32.7 ± 0.38	95.72 ± 3.91

**Table 4:** Composition of checkpoint batch along with predicted and observed values.

Checkpoint Batch	X_1_	X_2_	Gelation Temp (°C)		% Drug Release after 8 h	
	0.27%	19.8%	Predicted value	Observed value	Predicted value	Observed value
			30.39	30.5	95.28	94.36

**Table 5:** Pharmacokinetic parameters of darunavir in brain and plasma after administration by nasal (optimized in situ gel, 30 µL, 2.4 mg/kg) and intravenous (solution, 0.4 mL, 2.4 mg/kg) routes in Sprague Dawley rats. The data presented are the average of six animals at every time point.

Parameters	Brain		Plasma	
	Nasal	Intravenous	Nasal	Intravenous
T_max_^a^	1 h	2 h	2 h	-
C_max_^b^	260.02 ± 17.71 * (ng/g)	70.10 ± 5.07 (ng/g)	64.99 ± 14.28 * (ng/mL)	390.59 ± 32.61 (ng/mL)
AUC_0-α_^c^	1127.08 ± 267.72 * (ng·h/g)	325.84 ± 88.21 (ng·h/g)	414.25 ± 128.87 * (ng·h/mL)	1403.39 ± 303.82 (ng·h/mL)
AUC brain/AUC plasma	2.72	0.23	-	-

* Significant difference (*p* < 0.0001) detected in darunavir level in the nasal group compared to intravenous. ^a^ time to reach maximum plasma concentration; ^b^ maximum plasma drug concentration; ^c^ area under the plasma drug concentration–time profile curve.

**Table 6:** The layout of 3^2^ full factorial designs.

Experimental Layout of Design Batches					
	Coded Variables		Actual Variables		
Batch	X_1_	X_2_	Carbopol 934P Concentration	Poloxamer 407 Concentration	
D1	−1	0	0.2%	20%	
D2	+1	0	0.4%	20%	
D3	−1	−1	0.2%	19%	
D4	−1	+1	0.2%	21%	
D5	+1	−1	0.4%	19%	
D6	+1	+1	0.4%	21%	
D7	0	0	0.3%	20%	
D8	0	+1	0.3%	21%	
D9	0	−1	0.3%	19%	
Independent variables			Coded values	Actual values	
				X_1_	X_2_
X_1_ = Carbopol 934P concentration			−1	0.2%	19%
			0	0.3%	20%
X_2_ = Poloxamer 407 concentration			1	0.4%	21%

## Data Availability

The data presented in this study are contained within the article.

## Supplementary Materials

The following supporting information can be downloaded at: https://www.mdpi.com/article/10.3390/gels8060342/s1, Figure S1: Comparison of percentage darunavir release from in situ gels (F1–F9). The data presented are the average ± SD (n = 6).; Table S1: Phase transition temperature study of Poloxamer 407 solutions; Table S2: Model fitting for selected in situ gel (D7).
