# Supplementary material for: Intranasal Delivery of Darunavir-Loaded Mucoadhesive In Situ Gel: Experimental Design, In Vitro Evaluation, and Pharmacokinetic Studies
Source: Gels. 2022 May 30;8(6):342. doi: 10.3390/gels8060342 (PMC9223067; doi:10.3390/gels8060342)
Supplement: Supplementary file 1 [file gels-08-00342-s001.zip › gels-1733368-supplementary.pdf]

Table S1. Phase transition temperature study of Poloxamer 407 solutions.

Poloxamer 407 Concentration (% w/v)	Transition Temperature (°C)		Observation
	At Initial (Liquid) Stage	At Gelling Stage	
15	4	-	No phase transition took place till 40 °C.
16	4	-	No phase transition took place till 40 °C.
17	4	-	Viscosity increases at 38 °C but no phase transition took place till 40 °C.
18	4	37.04 ± 0.37	Gelled at 37 °C–38 °C
19	4	35.44 ± 0.52	Gelled at 35 °C–36 °C
20	4	33.18 ± 0.18	Gelled at 33 °C–34 °C
21	4	30.42 ± 0.21	Gelled at 30 °C–31 °C
22	4	26.75 ± 0.23	Gelled at 26 °C–27 °C
23	4	22.57 ± 0.16	Gelled at 22 °C–23 °C
24	4	19.80 ± 0.11	Gelled at 19 °C–20 °C
25	4	15.42 ± 0.37	Gelled at 15 °C–16 °C

Table S2. Model fitting for selected in situ gel (D7).

Model Name	Multiple R	r2	X Variable	Slope	SSR	Fischer Ratio
Zero order	0.9870	0.9741	11.4547	9.6988	209.4831	29.9262
First order	0.9752	0.9510	−0.1455	2.0926	887.1676	126.7382
Higuchi	0.9880	0.9762	34.9555	−7.8141	192.1643	27.4520
Korsmeyer – Peppas	0.9987	0.9974	0.7001	−0.6480	20.4716	2.9245
Weibull Model	0.9833	0.9669	1.1425	−0.6676	128.0530	18.2933
Hixson – Crowell	0.9947	0.9894	0.3453	−0.0610	100.3033	14.3290

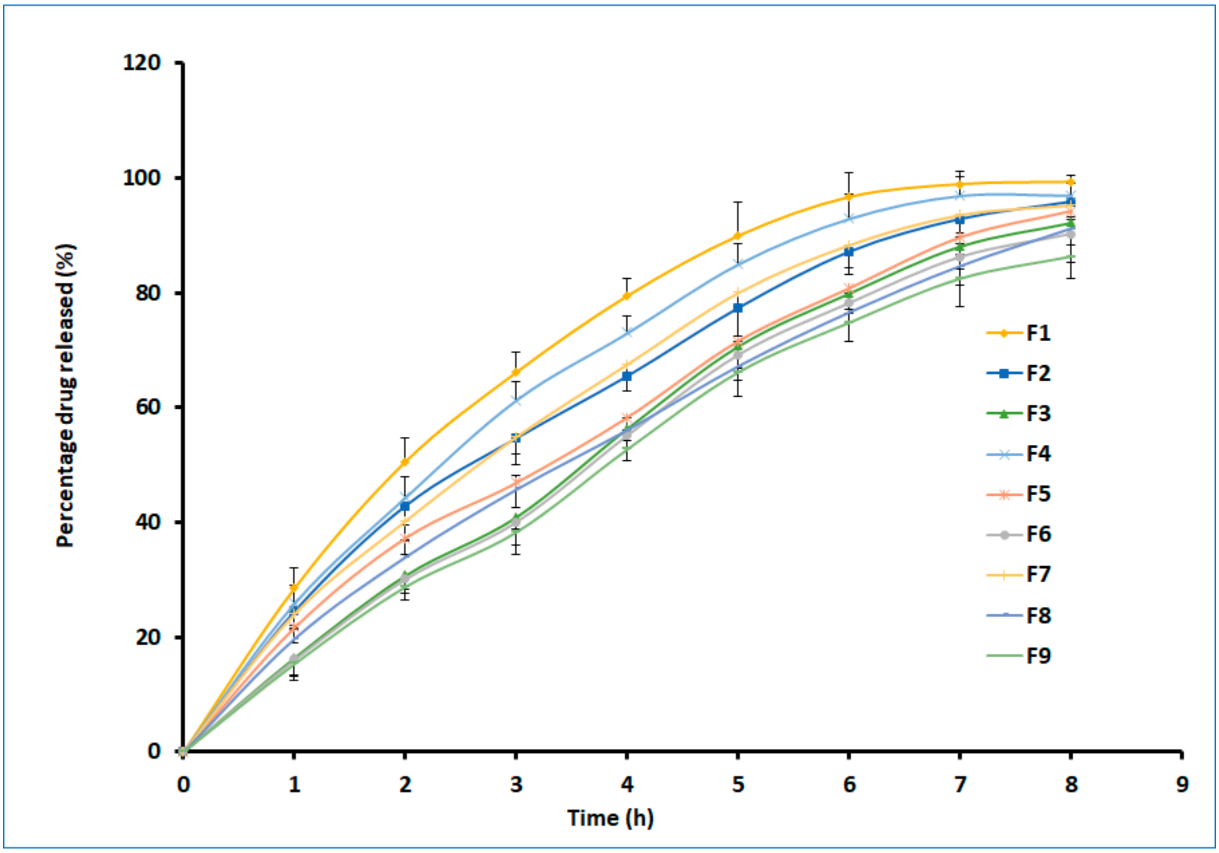

Figure S1. Comparison of percentage darunavir release from in situ gels (F1-F9). The data presented are the average ± SD (n = 6).
